# Association Between Obesity and Post-COVID-19 Condition in Military Conscripts

**DOI:** 10.3390/jcm15010355

**Published:** 2026-01-03

**Authors:** Reinhard Domanyi, Emanuel Maitz, Alexandros Andrianakis

**Affiliations:** 1 Military Hospital Graz, 8052 Graz, Austria; 2 Department of Otorhinolaryngology, Medical University of Graz, 8010 Graz, Austria

**Keywords:** adiposity, overweight, body mass index, BMI, waist-to-height ratio, long COVID

## Abstract

**Objectives:** Obesity has been suggested as a possible risk factor for the post-COVID-19 condition, but most studies rely only on body mass index (BMI), which does not reflect body fat distribution. Waist-to-height ratio (WHtR) is a simple anthropometric indicator of central obesity and a practical proxy for body fat distribution, yet it has not been studied in relation to the post-COVID-19 condition. This study aimed to examine whether obesity, measured by BMI and WHtR, is associated with the post-COVID-19 condition. **Methods:** A total of 500 male military conscripts (aged 18 years) underwent anthropometric measurements (height, weight, and waist circumference). Participants with prior COVID-19 were asked whether they had persistent or new symptoms after infection. BMI categories followed WHO definitions, and WHtR ≥ 0.50 was used to define central obesity. **Results:** Of the 376 participants who had previously experienced COVID-19, 82 (21%) experienced the post-COVID-19 condition. Obesity (BMI ≥ 30) was more common among those with the post-COVID-19 condition than those without (15% vs. 5%). BMI-defined obesity was associated with higher odds of the post-COVID-19 condition (OR 2.80, 95%CI 1.25–6.24). Central obesity was also more frequent in the post-COVID-19 condition (26% vs. 14%) and was linked to increased odds as well (OR 2.18, 95% CI 1.20–3.97). **Conclusions:** Both BMI-defined obesity and central obesity were associated with the post-COVID-19 condition. While WHtR does not directly quantify body fat distribution, it represents a simple and feasible anthropometric indicator. Therefore, it may be an additional useful tool for identifying individuals at higher risk of prolonged symptoms after COVID-19 infection.

## 1. Introduction

Infection with SARS-CoV-2 leads to an acute multisystem disease referred to as coronavirus disease 2019 (COVID-19) [1]. Over the past few years, it has become increasingly evident that a substantial proportion of individuals with COVID-19 continue to experience persistent symptoms following the resolution of the acute infection. This condition, commonly referred to as the post-COVID-19 condition or long COVID, is recognized by the World Health Organization (WHO) as a multisystem disorder that lasts for at least two months and cannot be explained by alternative diagnoses [2]. The post-COVID-19 condition has been linked to a wide variety of symptoms and health consequences [3,4,5]. Systematic reviews consistently highlight fatigue, breathing difficulties/chest tightness, muscle and joint pain, headaches, chest pain, impaired smell and taste, cognitive dysfunction, and gastrointestinal issues as the most prevalent symptoms, all of which can significantly impair daily activities, productivity, and quality of life [6,7,8]. The post-COVID-19 condition has received increasing attention in both clinical practice and research settings. Although most research has focused primarily on hospitalized and older adults, studies have shown that young people with mild acute disease can also develop long-lasting symptoms [9,10]. Importantly, the post-COVID-19 condition in young adults may have long-term consequences for physical performance, education, and work ability.

Understanding risk factors is essential not only for clinical management but also for prevention and early intervention strategies. Several demographic and clinical factors have been identified as potential risk factors for the post-COVID-19 condition. Higher age, female sex, severe acute infection requiring intensive care, and pre-existing conditions like cardiovascular disease or diabetes have consistently been associated with an increased risk of persistent symptoms after infection. In addition, obesity has also emerged as a strong risk factor for the post-COVID-19 condition [10,11,12]. Several biological mechanisms have been proposed to explain this association, particularly in the context of central adiposity. Visceral fat contributes to chronic low-grade inflammation and altered adipokine signaling, which may disrupt immune regulation and delay recovery after acute infection. Obesity is also linked to endothelial and microvascular dysfunction, a prothrombotic state, and impaired tissue oxygenation, all of which have been implicated in the pathophysiology of the post-COVID-19 condition [13].

A higher body mass index (BMI) has been associated with an increased likelihood of persistent symptoms after COVID-19 in several epidemiological studies [14,15,16,17]. However, the BMI has well-known limitations: it does not distinguish between fat and lean mass and provides no information on how body fat is distributed. Although advanced techniques such as bioelectrical impedance analysis or dual-energy X-ray absorptiometry provide precise assessment of body composition, they have not been applied in epidemiological studies of the post-COVID-19 condition, likely due to limited feasibility and cost. Evidence on anthropometric measures beyond BMI remains scarce. To date, only a single study has examined waist circumference (WC) as a marker of central obesity in relation to the post-COVID-19 condition. The Isfahan COVID cohort study reported an association between increased WC and the post-COVID-19 condition [18]. However, WC alone does not account for differences in body size or stature, which may limit its comparability across individuals. Current clinical guidelines therefore recommend including waist-to-height ratio (WHtR) when assessing obesity [19,20,21], as WHtR is a more sensitive marker of central fat accumulation and cardiometabolic risk than WC and BMI [22]. Nevertheless, the association between WHtR and the post-COVID-19 condition has not yet been systematically investigated.

Austria’s mandatory military induction examination offers an ideal framework for investigating this issue in a standardized and population-based manner. All 18-year-old male conscripts undergo uniform assessment conducted by trained professionals, enabling the collection of objective anthropometric measurements, including body height, weight, and waist circumference. This allows for the accurate calculation of both BMI and WHtR, ensuring a reliable assessment of general and central obesity.

This cross-sectional study aimed to determine the prevalence of the post-COVID-19 condition in male conscripts in Austria and to evaluate whether obesity—assessed using BMI and WHtR—is associated with a higher likelihood of the post-COVID-19 condition.

## 2. Materials and Methods

### 2.1. Study Design and Setting

This cross-sectional study was conducted at the Austrian Military Induction Board in Graz, Austria, between May 2024 and June 2025. The induction board serves as the national assessment center for all male conscripts entering mandatory military or community service at the age of 18 years in Austria. During the study period, all eligible conscripts undergoing their mandatory examination were invited to participate. The aim of this study was to examine the association between obesity, assessed by BMI and WHtR, and the post-COVID-19 condition in a representative sample of young adults.

### 2.2. Participants

A total of 500 male conscripts, all aged 18 years, were enrolled consecutively during their mandatory induction examination. Participants were included systematically as they presented for examination. The inclusion criteria were attendance at the military induction assessment and the provision of written informed consent to participate. No other eligibility criteria were applied as the study sought to reflect a population-based sample of young Austrian adults. All participants were free of acute illness at the time of examination. Participants without a history of COVID-19 infection were included only for a descriptive characterization of the total cohort and were not included in any analyses related to the post-COVID-19 condition.

### 2.3. Data Collection

For each participant, a history of confirmed COVID-19 infection was collected retrospectively based on self-reporting measures. Participants with previous COVID-19 infections were then asked if they had experienced long-lasting, persistent, or new symptoms following the infection. Body weight, height, and WC were measured using uniform procedures by trained staff during the military induction examination, to ensure standardized anthropometric assessment. Participants were measured in their underwear and without shoes. WC was measured using a non-elastic tape, with participants standing upright and breathing normally. BMI was calculated as weight in kilograms divided by height in meters squared (kg/m^2^). Based on the WHO classification, participants were categorized as underweight (BMI < 18.5 kg/m^2^), normal weight (18.5–24.9 kg/m^2^), overweight (25.0–29.9 kg/m^2^), or obese (≥30.0 kg/m^2^) [23]. WC was measured midway between the lowest rib and the iliac crest at the end of normal expiration, according to the WHO protocol [24]. Based on the IDF criteria for metabolic syndrome, a WC ≥ 94 cm was defined as central obesity [25]. The WHtR was derived by dividing WC (cm) by body height (cm). A WHtR ≥ 0.50 was considered as central obesity, according to the current guidelines [19,20,21].

### 2.4. Statistical Analyses

The SPSS © statistical software, version 29.0 (IBM ©, Armonk, NY, USA), was used for statistical analysis. Continuous variables were reported as means ± standard deviations. Categorical variables were presented as absolute numbers and percentages. Chi-square tests were used to compare unpaired categorical variables. In contingency tables with more than two categories per variable (BMI), Bonferroni-adjusted column proportion Z-tests were applied as post hoc procedures to identify specific subgroup differences. Binary logistic regression analyses were performed to quantify the associations between BMI categories and the post-COVID-19 condition, using normal weight as the reference category, as well as WHtR and the post-COVID-19 condition (WHtR < 0.5 vs. ≥0.5). BMI and WHtR were analyzed in separate models to avoid collinearity and overadjustment, as both measures capture related aspects of adiposity. Odds ratios (ORs) with 95% confidence intervals (CIs) were reported. The statistical significance level was set to a *p*-value of <0.05, two-sided.

### 2.5. Ethical Considerations

The study was conducted in accordance with the ethical guidelines of the Declaration of Helsinki and approved by the institutional Ethics Committee of the Medical University of Graz for studies involving humans (approval code: 36-090 ex23/24; date of approval: 8 March 2024). All participants involved in this study provided written informed consent.

## 3. Results

### 3.1. Clinical Characteristics of the Total Cohort and Prevalence of the Post-COVID-19 Condition

A total of 500 male subjects, aged 18 years, were included in this study. Of these, 376 subjects (75%) had previous confirmed COVID-19 infections. Table 1 summarizes the clinical characteristics of the entire cohort and the subgroups with and without previous infection. Anthropometric parameters were comparable between individuals with and without a history of COVID-19 (all *p* > 0.05). Among the 376 participants with prior COVID-19, 82 individuals (21%) had the post-COVID-19 condition. All subsequent analyses examining the post-COVID-19 condition were restricted to participants with a prior history of COVID-19 infection.

### 3.2. Body Mass Index and the Post-COVID-19 Condition

Within participants with a prior history of COVID-19 infection, BMI categories were distributed as follows: underweight for 23 participants (6%), normal weight for 243 (65%), overweight for 81 (22%), and obesity for 29 (8%). While the overall chi-square test across all four BMI categories was not significant (*p* = 0.066), Bonferroni-adjusted column proportion tests indicated that the obesity category was significantly more common among individuals with the post-COVID-19 condition than among those without (15% vs. 5%). Detailed results are presented in Table 2.

### 3.3. Waist Circumference and the Post-COVID-19 Condition

Among participants with previous COVID-19 histories, 38 individuals (10%) had increased WCs, indicative of central obesity. Central obesity defined by WC was significantly more prevalent in individuals with the post-COVID-19 condition than in those without (18% vs. 7%; *p* = 0.008). There were no significant differences in body height between individuals with and without the post-COVID-19 condition (*p* = 0.847). Detailed results of the WC comparisons are shown in Table 3.

### 3.4. Waist-to-Height-Ratio and the Post-COVID-19 Condition

Among participants with previous COVID-19 histories, 61 individuals (16%) demonstrated central obesity based on a WHtR ≥ 0.50. Central obesity was significantly more frequent among individuals with the post-COVID-19 condition (26%) than in those without (14%) (*p* = 0.012). Detailed comparison data of WHtR categories between the post-COVID groups is presented in Table 4.

### 3.5. Logistic Regression Analyses Predicting the Post-COVID-19 Condition

To further assess the association between obesity and the post-COVID-19 condition, logistic regression models were performed using BMI categories and WHtR. Both parameters were analyzed in separate models to avoid collinearity and overadjustment, as both measures capture related aspects of adiposity. The results showed that BMI-defined obesity (BMI ≥ 30) and central obesity, assessed by WHtR (WHtR ≥ 0.50), were both significantly associated with increased odds of the post-COVID-19 condition. The detailed results are presented in Table 5.

## 4. Discussion

This study adds novel evidence to the growing body of literature on obesity as a risk factor for the development of the post-COVID-19 condition. In a population-based cohort of young adult male conscripts, we observed that both BMI-classified obesity and central adiposity defined by WtHR ≥ 0.50 were significantly associated with the post-COVID-19 condition. Importantly, the association with WHtR suggests that body fat distribution, and not only body weight, may be relevant for the persistence of symptoms after COVID-19.

Obesity has become a major global health burden, affecting populations across all regions and age groups. Overweight and obesity are key contributors to numerous non-communicable diseases—including cardiovascular, musculoskeletal, metabolic, and respiratory conditions—and are strongly linked to premature death. According to recent WHO estimates, 2.5 billion adults aged over 18 years were overweight in 2022, corresponding to a global prevalence of 43% [26]. In Europe, 20.6% of individuals aged 16–24 years were overweight or obese in 2022, with rates of 26% in Austria [27]. In our cohort, 28% of Austrian 18-year-old male conscripts were overweight or obese, reflecting the national estimates and underscoring the representativeness of our sample.

Obesity has increasingly been recognized as an important risk factor for the post-COVID-19 condition. Several large epidemiological studies have consistently reported a higher likelihood of persistent symptoms among individuals with elevated BMI following COVID-19 infection [14,15,16,17]. Our findings are in line with this evidence and further show that this association is already present at a young age. In our cohort, obesity was nearly three times more prevalent among participants with the post-COVID-19 condition than among those without persistent symptoms, and logistic regression confirmed the significantly increased odds of the post-COVID-19 condition among individuals with BMI-defined obesity. In contrast, underweight and overweight categories were not associated with the post-COVID-19 condition, suggesting that more pronounced adiposity may be required to substantially increase risk. However, the BMI has important limitations: it cannot distinguish between fat and lean mass and offers no information about how adipose tissue is distributed in the body. This limitation is particularly relevant in young adults, in whom a higher BMI may reflect greater muscle mass rather than excess fat. Central obesity, which reflects visceral fat accumulation, is more closely linked to metabolic dysfunction and inflammation than total body weight [13]. Only limited evidence exists on anthropometric measures of central adiposity beyond BMI in relation to the post-COVID-19 condition. To date, a single population-based study has examined WC as an indicator of central obesity in this context. The Isfahan COVID cohort study showed that individuals with overweight and increased WC had a significantly higher risk of the post-COVID-19 condition [18]. We observed similar findings in our cohort. Participants with a WC indicative of central obesity were significantly associated with the post-COVID-19 condition. Nevertheless, as WC has been used as indicator for abdominal obesity, it does not account for differences in body size or stature and may therefore be less suitable for risk stratification. In this context, the WHtR has important practical advantages. By standardizing WC for height, WHtR provides a more robust estimate of central adiposity and has consistently outperformed both BMI and waist circumference in predicting cardiometabolic risk [22]. Due to its superiority, current clinical guidelines therefore included WHtR as the recommended anthropometric measure for diagnosing obesity [19,20]. Despite this, WHtR has not previously been studied in relation to the post-COVID-19 condition. To the best of our knowledge, this is the first study to address this knowledge gap. In our cohort, central obesity, defined by a WHtR ≥ 0.50, was almost twice as common among individuals with the post-COVID-19 condition compared with those without persistent symptoms. Logistic regression further supported this association: participants with a WHtR ≥ 0.50 had more than double the odds of developing the post-COVID-19 condition. These findings suggest that WHtR may capture obesity-related risks for the post-COVID-19 condition more effectively than BMI alone. Given its simplicity and strong predictive value, WHtR may represent a practical anthropometric tool for identifying young adults who are more vulnerable to prolonged recovery after COVID-19 infections. These findings are particularly relevant in the context of increasing obesity prevalence at young ages. Early adulthood represents a critical period for long-term health, and prolonged symptoms after COVID-19 may interfere with education, employment, and physical activity.

Several mechanisms may explain why obesity increases the risk of the post-COVID-19 condition [13]. Excess abdominal fat can mechanically restrict lung expansion and diaphragmatic movement, potentially contributing to persistent respiratory complaints [28]. Visceral fat is metabolically active and promotes chronic low-grade inflammation through an increased production of pro-inflammatory cytokines such as IL-6 and TNF-α. This inflammatory state may impair immune regulation and delay recovery after acute COVID-19 infection [29,30]. Moreover, obesity is associated with a higher risk of vascular injury. Lipid accumulation can weaken the vascular endothelium, and the formation of microthrombi may irritate the vessel wall and promote aseptic inflammation [31]. Adipose tissues may also serve as a potential site where viruses persist longer, which could maintain immune activation [32,33]. Together, these mechanisms provide biological plausibility for the observed association between obesity and the post-COVID-19 condition.

An important strength of this study is the focus on a homogeneous cohort of young adult men. The post-COVID-19 condition is often considered to be a problem mainly affecting middle-aged or older adults. However, our findings demonstrate that obesity-related risks are present even in young individuals. This suggests that metabolic health may influence recovery independently of age and highlights central obesity as a potential marker of reduced physiological resilience.

However, the are some limitations that must be acknowledged. First, the cohort consisted exclusively of 18-year-old male Austrian conscripts, which limits generalizability to females, older adults, and broader populations. Second, the histories of COVID-19 infection and post-COVID-19 symptoms were based on self-reporting measures, which may introduce recall bias and misclassification. Third, socio-demographic characteristics and pre-existing physical or mental health conditions that may influence both obesity status and the risk of the post-COVID-19 condition were not systematically assessed and could therefore not be adjusted for in the analyses. Fourth, although obesity was measured directly through recommended anthropometric assessments by trained staff—a strength of this study—we did not use more precise methods to quantify body fat or visceral adiposity, such as bioelectrical impedance analysis, dual-energy X-ray absorptiometry, or imaging techniques. These tools could have provided more detailed information about fat distribution and metabolic risk. Finally, the cross-sectional, observational design does not allow for conclusions to be made regarding direct causality.

Future studies should use longitudinal designs to better understand how obesity influences the development and persistence of the post-COVID-19 condition over time. In addition to simple anthropometric measures, future research should incorporate objective assessments of obesity and body composition, such as imaging- or impedance-based methods, together with evaluations of physical performance, inflammatory markers, and cardiopulmonary function, to better clarify underlying biological mechanisms. Furthermore, studies in more diverse populations, including women and older adults, are also needed to confirm the broader generalizability of these findings. Finally, it will be important to examine whether lifestyle interventions, weight reduction, or improvements in metabolic health can positively influence recovery and reduce the risk of the post-COVID-19 condition.

## 5. Conclusions

Our findings suggest that both general obesity and central adiposity are associated with a higher likelihood of the post-COVID-19 condition among young adult men with prior COVID-19 infections. Because WHtR is simple to measure and reflects central adiposity more accurately than BMI, it may serve as a useful additional marker. As central obesity continues to rise in adolescents and young adults, incorporating WHtR into routine health assessments may help identify individuals who are at greater risk of prolonged post-infectious symptoms.

## Figures and Tables

**Table 1:** Anthropometric measures of the total cohort, stratified by history of COVID-19 infection.

Parameter	Total Cohort (N = 500)	History of COVID-19 Infection (n = 376, 75%)	No COVID-19 Infection (n = 124, 25%)
Body height (cm)	178 ± 6	178 ± 6	178 ± 6
Body weight (kg)	75.7 ± 14.5	74.8 ± 12.8	76.0 ± 15.1
Waist circumference (cm)	79.8 ± 10.3	80.0 ± 10.8	79.4 ± 8.7
BMI (kg/m^2^)	23.7 ± 4.1	23.5 ± 3.7	23.8 ± 4.3
Underweight (BMI < 18.4)	32 (6%)	23 (6%)	9 (7%)
Normal weight (BMI 18.5–24.9)	327 (65%)	243 (65%)	84 (68%)
Overweight (BMI 25–29.9)	104 (21%)	81 (21%)	23 (18%)
Obesity (BMI ≥ 30)	37 (7%)	29 (8%)	8 (6%)
Healthy WtHR (<0.5)	420 (84%)	315 (84%)	105 (85%)
Central Obesity (WtHR ≥ 0.5)	80 (16%)	61 (16%)	19 (15%)

**Table 2:** BMI categories among participants with and without the post-COVID-19 condition.

BMI Category	Post-COVID-19 (n = 82)	No Post-COVID-19 (n = 294)	*p* = 0.066
Underweight (BMI < 18.4)	4 (5%) _a_	19 (7%) _a_	
Normal weight (BMI 18.5–24.9)	49 (60%) _a_	194 (66%) _a_	
Overweight (BMI 25–29.9)	17 (20%) _a_	64 (22%) _a_	
Obesity (BMI ≥ 30)	12 (15%) _a_	17 (5%) _b_	

Each subscript letter (a, b) denotes a subset of post-COVID-19 groups whose proportions do not differ significantly from each other at the Bonferroni-adjusted significance level.

**Table 3:** Comparison of waist circumference between participants with and without the post-COVID-19 condition.

Parameter	Post-COVID-19 (n = 82)	No Post-COVID-19 (n = 294)	
Waist circumference			*p* = 0.008
Healthy (<94 cm)	67 (82%)	271 (93%)	
Central obesity (≥94 cm)	15 (18%)	23 (7%)	

**Table 4:** Comparison of waist-to-height ratio between participants with and without the post-COVID-19 condition.

Parameter	Post-COVID-19 (n = 82)	No Post-COVID-19 (n = 294)	
Waist-to-Height Ratio			*p* = 0.012
Healthy (<0.5)	61 (74%)	254 (86%)	
Central Obesity (≥0.5)	21 (26%)	40 (14%)	

**Table 5:** Association between BMI categories, WHtR, and the post-COVID-19 condition: logistic regression results.

Predictor	Odds Ratio (OR)	95% CI	*p*-Value
Underweight (BMI < 18.4)	0.83	0.27–2.56	0.751
Overweight (BMI 25–29.9)	1.05	0.57–1.96	0.873
Obesity (BMI ≥ 30)	2.80	1.25–6.24	0.012
Central obesity (WHtR ≥ 0.50)	2.18	1.20–3.97	0.010

Normal weight (BMI 18.5–24.9) and WHtR < 0.50 served as reference categories. BMI categories and WHtR were analyzed in separate logistic regression models.

## Data Availability

The original contributions presented in the study are included in the article; further inquiries can be directed to the corresponding author.
